# New Appliances and Things Medical

**Published:** 1897-05-15

**Authors:** 


### NEW APPLIANCES AND THINGS MEDICAL.

[We shall be glad to receive, at our Office, 28 & 29, Southampton Street, Strand, London, W.C., from the manufacturers, specimens of all new preparations and appliances which may be brought out from time to time.]

### SALUTARIS WATER.

(236, Fulham Road, S.W.)

Salutaris water is one of the few really pure waters on the London market. Many popular brands are relatively pure when compared with ordinary drinking water, but no water that is not the result of artificial distillation can be regarded as absolutely pure. As is well known, Salutaris water is manufactured by a special process of distillation, and condensation of the resulting steam. Simple distilled water is sweetish in taste and decidedly flat, but when charged with gas under high pressure it becomes sparkling and invigorating. This is fully exemplified in the case of Salutaris, which is highly charged with carbonic acid gas under pressure amply sufficient to render it one of the most sparkling of the aërated waters. It is consequently entirely free from foreign matter, organisms, and germs. In drinking Salutaris one may rest assured that nothing but the pure oxide of hydrogen and carbonic acid enter the system. Being tasteless and unimpregnated with salts, it lends itself most happily to admixture with spirits, and for ordinary table purposes it is unequalled.

### THE STETHONOSCOPE.

(S. Maw, Son, and Thompson, 7 to 12, Aldersgate Street, E.C.)

This new development of the binaural stethoscope offers considerable advantages in the auscultation of pulmonary and cardiac sounds. Being constructed somewhat on the principle of the microphone it has the power of intensifying or rendering audible sounds which might otherwise escape the notice of the physician. We have made practical trial of the stethonoscope during the last six weeks, and can assure our readers that in ordinary practice it has proved itself a most useful adjunct to the usual methods of diagnosis. In private practice the conditions are seldom such as to offer the best opportunities for diagnosis, and to obtain a clear and distinct hearing of the often obscure sounds of the chest is of the greatest advantage. The instrument is by no means complicated, and can be readily carried in the waistcoat pocket without damage. The only practical disadvantage that we have so far discovered is that the various parts of which it is composed too readily become separated, and the tap which regulates the amount of sound reaching the ear is apt to become lost by reason of its small size. The stethonoscope can be applied to the rubber tubes of an ordinary binaural stethoscope and used with the ear pieces. We believe that this new improvement in auscultating methods will be very generally adopted.

### AN AUTOMATIC SPRING TONSIL GUILLOTINE.

(Arnold and Sons, West Smithfield.)

The present form of tonsil guillotine is really a modified form of that introduced by the late Sir Morell Mackenzie. The difference consists in the adaptation of a spring between the knife slide and the holder. The spring is held and released by means of a trigger. The instrument can be easily taken to pieces and the spring removed for cleaning purposes. The advantages of this new guillotine are: (1) Rapidity of action; (2) the ease with which it can be worked with one hand, leaving the other free for other purposes; (3) simplicity of structure; (4) certainty of action. The modification is due to Mr. R. Brooks Popham.

